# *BRCA1* Haploinsufficiency Leads to Altered Expression of Genes Involved in Cellular Proliferation and Development

**DOI:** 10.1371/journal.pone.0100068

**Published:** 2014-06-20

**Authors:** Harriet E. Feilotter, Claire Michel, Paolo Uy, Lauren Bathurst, Scott Davey

**Affiliations:** 1 Departments of Pathology and Molecular Medicine, Queen's University, Kingston, Ontario, Canada; 2 Departments of Biomedical and Molecular Sciences and Oncology, Queen's University, Kingston, Ontario, Canada; 3 Cancer Research Institute, Queen's University, Kingston, Ontario, Canada; 4 Division of Cancer Biology and Genetics, Queen's University, Kingston, Ontario, Canada; Cnr, Italy

## Abstract

The assessment of BRCA1 and BRCA2 coding sequences to identify pathogenic mutations associated with inherited breast/ovarian cancer syndrome has provided a method to identify high-risk individuals, allowing them to seek preventative treatments and strategies. However, the current test is expensive, and cannot differentiate between pathogenic variants and those that may be benign. Focusing only on one of the two BRCA partners, we have developed a biological assay for haploinsufficiency of *BRCA1*. Using a series of EBV-transformed cell lines, we explored gene expression patterns in cells that were *BRCA1* wildtype compared to those that carried (heterozygous) *BRCA1* pathogenic mutations. We identified a subset of 43 genes whose combined expression pattern is a sensitive predictor of *BRCA1* status. The gene set was disproportionately made up of genes involved in cellular differentiation, lending credence to the hypothesis that single copy loss of BRCA1 function may impact differentiation, rendering cells more susceptible to undergoing malignant processes.

## Introduction

Breast cancer is one of the most common forms of cancer, and one of the leading causes of cancer-related deaths throughout the western world. While the majority of breast cancer cases are sporadic, 5–10% are classified as hereditary, and are due to the presence of a mutation in a breast cancer predisposition gene [1]. Approximately half of all hereditary breast cancers are due to a mutation in either *BRCA1* or *BRCA2*, and approximately 80% of individuals with a mutation in either of these genes develop breast cancer by the age of 70 years [2]. Because of the high risk of cancer in individuals with these mutations, their early and accurate identification targets them for increased surveillance and/or protective interventions such as surgery.

However, the task of identifying carriers of *BRCA* mutations is complicated by our continued lack of understanding of the specific biological mechanisms that are impacted by mutation of either gene. Additionally, the consistent evaluation of both *BRCA1* and *BRCA2* as a gene set implies that functional assays must necessarily be broad enough to capture activities of both proteins, an unreasonably difficult task. Therefore, we have chosen to focus on each of the genes as an independent problem to highlight the approach for development of a biological assay to explore loss of a complex protein. The work described here is focused on measurement of loss of *BRCA1* function.

Since the identification of *BRCA1* almost two decades ago [3], the molecular pathways in which BRCA1 functions and how disruptions of these functions promote breast and ovarian carcinogenesis remains a mystery. The human *BRCA1* gene encodes an 1863 amino-acid protein, containing two highly conserved domains in the N- and C-terminal regions of the protein. At the N-terminus lies a RING domain, a cysteine rich zinc-binding motif that functions as an E3 ligase enzyme involved ubiquitination [4]. Two tandem repeat globular domains termed BRCT, a common feature of proteins involved in the DNA damage repair and cell cycle control [5], lie at the C-terminus. Functionally, BRCA1 has been implicated in a diverse array of cellular functions, including ubiquitination [6]–[9], regulation of the G1/S [10], intra-S and G2/M-phase cell cycle checkpoint control [11]–[14], regulation of spindle pole body duplication[15], transcription [16]–[19], sex chromosome inactivation [20]–[23] and homologous recombination repair of double stranded DNA breaks [24], [25]. Taken together, these individual roles suggest a function for BRCA1 in the maintenance of genomic integrity [26], [27]. BRCA1 has also been suggested to a play a role in the differentiation of breast epithelial cells, with loss of BRCA1 function leading to impaired acini formation in 3D culture and an accumulation of less differentiated cells with altered proliferation properties [28]–[31].

Current methods for the identification of *BRCA1* carriers are based on gene sequence variations. One of the inherent difficulties in this approach is in differentiating between clinically important changes and benign polymorphisms in these genes. While *BRCA1* mutations that result in a truncated protein can usually unequivocally be called disease-causing, many other mutations, termed variants of unknown significance (VUS) are more difficult to interpret in a clinical context. Approximately 13% of *BRCA1* and *BRCA2* genetic tests reveal mutations identified as VUSs [32].

Tracking how specific *BRCA1* mutations segregate with disease within families as well as case-control studies provide the most reliable information for classifying VUS as pathogenic or neutral. Case control studies are made difficult, however, by the rarity of specific mutations in the population, while segregation studies suffer from uncertainties generated by the high likelihood of phenocopies among the affected, and the potential for late onset cancer in the unaffected. In cases where clinical data are not available to classify VUS, several functional assays have been developed to assess the effects of individual mutations on specific BRCA1 functions, including a transcriptional activation assay [33], phosphopeptide binding assay [34], ubiquitin ligase activity assay [35] and an embryonic stem-cell based functional assay [36]. However these functional assays can be technically complex and are limited to mutations in specific domains impacting particular functions. Regardless of whether the “correct” function is targeted in such assays, the requirement to assay multiple complex biochemical functions precludes the use of this approach in the clinical laboratory setting.

Given the pleiotropic roles of BRCA1, and the potential for individual mutations to lead to tumorigenesis via different mechanisms, the development of a functional assay for BRCA1 presents a significant challenge. However, because BRCA1 evidently plays a central role in many critical pathways that converge on the maintenance of genomic integrity, we hypothesized that there was a high likelihood that loss of even a single copy of *BRCA1* would have a measurable impact on the expression of downstream genes involved in one or more of these pathways. Indeed, *BRCA1* haploinsufficiency has been shown in several studies to alter the differentiation and proliferation pathways of breast epithelial/progenitor cells in patients carrying a *BRCA1* mutation [30], [31], [37].

Evidence lending support to the idea of carrier-phenotype expression profiling comes from several studies, including small-scale studies on *BRCA1* carrier fibroblasts following exposure to ionizing radiation (IR) [38], [39]. Bellacosa et al. 2010 [40], showed that *BRCA1* carriers had altered gene expression profiles in cultured primary breast and ovarian epithelial cells compared to non-carriers. Another recent study [41] showed that lymphocytes from *BRCA1* mutation carriers demonstrated altered gene expression profiles following exposure to IR, which could be used as a prediction tool to identify *BRCA1* mutation carriers. Here, we sought to determine whether EBV-transformed lymphoblastoid cell lines heterozygous for *BRCA1* mutations could be distinguished from control cell lines using whole genome gene expression profiling.

## Methods

### Samples

EBV-transformed lymphocytes (LCLs) were obtained through the NIH Breast Cancer Family Registries. The 69 cell lines used in this study included 38 control (*BRCA1^+/+^*) and 31 BRCA1 mutation carriers (*BRCA1^+/−^*). The carrier cell lines included frameshift, missense, nonsense, and splicing mutations; a list of the *BRCA1* mutations is shown in Table 1. All LCLs used in this study were cultured in RPMI-1640 media (Sigma Aldrich, Oakville, ON) supplemented with non-heat inactivated 15% fetal bovine serum (FBS) (Sigma Aldrich). All cell culture was carried out in 25 cm^2^ flasks (Corning, Nepean, ON) at 37°C in 5% CO_2_ atmosphere. Cells were split in a 2∶1 ratio until the desired cell number of 650,000 cells/ml was reached. Where noted, DNA damage was induced through exposure to 2 Gy ionizing radiation (IR), delivered by a ^137^Cs Victoreen Electrometer (Atomic Energy of Canada, Mississauga, ON) at a dose rate of 0.52 Gy/min. Following treatment, the cells were allowed to recover for a period of 6 hr at 37°C in 5% CO_2_ atmosphere prior to extraction of total RNA.

**Table 1:** List of BRCA1 mutations used in this study.

*ID*	*Mutation*	*Class*	*Test*
12928	c.4689C>G	N	
13135	c.66_67delAG	F	
13416	c.5263insC	F	
13537	c.3607C>T	N	Y
14023	c.2071delA	F	Y
14643	c.1175_1214del	F	Y
14663	c.4327C>T	N	
14703	c.2834_2836delGTAinsC	F	
14832	c.2475_2476delC	F	
14834	c.1016insA	F	
15268	c.5263insC	F	
15285	exon13ins6kb	O	
15736	c.191G>A	M	Y
15737	IVS1-22A>G	O	
16236	c.2561insGC	F	
17082	c.66_67delAG	F	Y
17653	c.5263insC	F	
18318	c.3756_3759delGTCT	F	
18700	IVS9-2A>C	S	
19018	c.4327C>T	N	
21303	c.66_67delAG	F	
22893	c.1175_1214del	F	Y
24262	c.851ins7	F	
25453	c.2934T>G	N	
26842	c.4327C>T	N	
26950	c.3695_3699del5	O	Y
27129	exon13 dup	F	
27131	c.4484G>T	M	Y
27348	c.66_67delAG	F	
27636	c.3607C>T	N	
33139	c.66_67delAG	F	

Class abbreviations are N: Nonsense; F: Frameshift; M: Missense; S: Splicing; O: Other. Test indicates the samples present in the test set; all other samples were used in the training set.

### Ethics Statement

This work was approved by the Queen's University Research Ethics Board under approval #PATH-115-10. Collection and generation of the LCL lines has been reported previously [42].

### Cell culture and gene expression profiling controls

To assess the characteristics of our samples prior to transcriptome analysis, we determined the kinetics of arrest and recovery (0–18 h) across a range of IR doses (0–4 Gy). In all cases, cells were exposed to Cell Proliferation Labeling Reagent (Amersham Biosciences, Baie d'Urfe, Canada), according to the manufacturers instructions, for 1 hour prior to harvest. A dose of 2 Gy followed by 6 hours of recovery was the minimum dose and maximum recovery time at which we observed a uniform G1-S arrest, as assayed by loss of the early S phase cells from 2 dimensional flow cytometry profiles. This dose/recovery scheme was used throughout the study. For all microarray experiments, two parallel cultures were generated and one was treated with 2 Gy IR, 6 hours prior to harvest. Only cell lines showing proliferation in untreated cells, G1 checkpoint arrest following IR, and producing high quality RNA were used for microarray analysis.

### Gene expression profiling

RNA from each of the 69 cell lines was extracted using TRIZOL Reagent following the manufacturer's recommendations (Invitrogen, Burlington, ON). RNA was purified using the RNeasy MinElute Cleanup Kit (Qiagen, Mississauga, ON). RNA quality was assessed by Agilent 2100 Bioanalyzer (Version B.02.02). RNA with an integrity number of at least 7 was amplified and labeled using the Agilent Low RNA Input Linear Amplification kit (Agilent, Santa Clara, CA). Labeling reactions were performed with 250 ng total RNA, along with the Agilent Spike-in RNA mix, using Cy3-CTP and Cy5-CTP for control (-IR) and experimental (+IR) RNA, respectively (Perkin Elmer, MA, USA). Amplified RNA was quantified using the NanoDrop ND-1000 (NanoDrop Technologies, DE, USA) and the concentration of cRNA and the specific dye activity were calculated. Samples with a specific dye activity greater than 8 pmol/µl were selected for hybridization to arrays. Pairs of cRNA (unirradiated versus irradiated) were hybridized to Agilent Whole Human Genome Oligo 4x44K GE arrays as per the product protocol. Image acquisition and analysis were done using an Agilent Microarray Scanner, Model G2565BA and Agilent Feature Extraction software v9.1 set to default settings. Raw data has been submitted to the NCBI GEO database (Accession Number GSE19541.)

### Data analysis: Non supervised clustering

(NSC) analysis was done using the PAM method [43] as a software add-on within Microsoft Excel. Heat maps of the final classifiers, normalized by chip, were constructed using the Genesis software package [44]. Pathway analysis was performed using the Ingenuity software package (www.ingenuity.com). Identification of radiation responsive genes was done using SAM [45].

For NSC analysis, a total of 43,338 features were used in the analysis. 38 features that had gene label values that could not be interpreted by the analysis software were eliminated from the dataset prior to analysis. Normalization, where used, is described in the relevant sections of the Results.

Comparisons between microarray and qPCR data were calculated as follows: For microarray data, log (2) ratios between the values for each sample was compared to the average of all cell lines. For qPCR samples, ΔCt values were calculated for each target gene relative to the GusB control, and ΔΔCt values were calculated relative to the ΔCt value of WT19998 as a control. Fold changes were calculated individually versus the average of all cell lines for which data were available. Average values were calculated independently for each of the BRCA1 haploinsufficient and WT cell lines.

## Results

### Basal gene expression levels can be used to distinguish *BRCA1^+/+^* from *BRCA1^+/−^* cells

Following the generation of whole transcriptome expression data from each of the logarithmically growing cell lines, we sought to determine whether gene expression values differed in *BRCA1^+/−^* versus *BRCA1^+/+^* lines. We used a nearest shrunken centroids analysis approach to analyze the data [43]. Data were analyzed either with no prior normalization, or following median normalization (per chip). These two approaches to analysis yielded similar and extensively overlapping results. However, the use of raw data tended to emphasize highly expressed genes, while the application of normalization algorithms permitted the identification of additional genes of interest that were expressed at lower levels. Therefore, we focused on the normalized datasets, although some genes unique to the non-normalized analysis were included in our final model.

We divided our samples into training (53 samples) and test (16 samples) randomly. Using the normalized data set, we observed the minimum training set error (6%, 3/53 samples) over a relatively wide range of shrinkage co-efficients (1.85–2.86), representing 288 to 23 genes, respectively. When this model was tested on the test set (8 wild type and 8 carriers), the accuracy was 95% (15/16), with a single misclassification of a control sample.

Modeling using the raw data was also effective, although it exhibited a slightly lower accuracy in the independent test set, and utilized a much larger number of genes in the predictor. The minimum training set error was 11% (6/53), and accuracy on the independent test set was 88% (14/16), with 2 control samples incorrectly classified, including the one that was previously misclassified using the model from the normalized data. The raw data model was accurate over a narrower range of shrinkage coefficients (1.35–1.65), representing 785 to 367 genes, respectively.

To generate a list of the most consistent predictive genes for biological analysis, we first selected genes that were identified by both approaches using the most stringent shrinkage coefficients (a total of 13 gene features). We then added genes that were ranked higher (by either model) than the lowest ranking of those identified by both approaches. In many cases, such genes were identified by both approaches if the shrinkage coefficients were relaxed to the lowest value that minimized training set error. A total of 17 genes were added by this approach. Finally, carrying out these approaches in both datasets (normalized or not) identified some genes were only identified in the raw (7 genes) or normalized (6 genes) data sets. The end result was a list of 43 genes, summarized in Table 2.

**Table 2:** List of genes identified as predicting BRCA1 carrier status.

*Gene*	*Notes*	*Predictive Value*	*PCR Validated*
PXDN	1	4.246558229	
JAKMIP2	4	2.147236883	
MMP7	2	2.062558949	
CSRP2	1	1.797239311	
CD24	1	1.75716275	
LFNG	2	1.706805758	
ENPP2	2	1.705982587	
FOXP1	1	1.503455224	
PWWP2	1	1.478776571	
PRLR	4	1.383493041	
IFNA5	4	1.228390716	
FCGRT	1	0.997556344	
IFNA4	1	0.99182988	
IFIT3	2	0.89448872	
SERPINF1	2	0.886742073	
IGHD	3	0.84862378	
IFIT1	2	0.788035184	Yes
ZBED3	2	0.786498412	
IFIT2	2	0.736255901	
USP18	3	0.727615739	
IFI44L	2	0.689378004	
SOX4	2	0.471075432	
MX2	3	0.468182764	
MX1	3	0.462444907	
HLA-DMB	3	−0.378283622	
DUSP23	2	−0.860935628	
GLDC	2	−0.878929021	
ZBTB38	2	−0.89526144	
BCR	2	−1.004874472	
LAG3	3	−1.068210822	
IL18BP	2	−1.173050671	
UBD	3	−1.174178777	
TNS4	4	−1.474875288	
SLC16A10	4	−1.558340909	
PLA2G4A	1	−1.62075284	
CYP1B1	2	−1.779383571	
FAM79B	1	−1.900252217	
IFNG	4	−1.97628822	
IGHG1	1	−2.110861375	Yes
FYN	1	−2.114129327	
CXCR3	1	−2.203429664	Yes
TBX21	1	−2.66823647	Yes
ETV7	2	−2.720734937	

The genes identified using the shrunken centroids analysis approach are listed. The list contains a total of 43 genes.

Notes: 1 means both from short list; 2 means both from long lists; 3 means raw only; 4 means normalized only.

**Figure 1:**
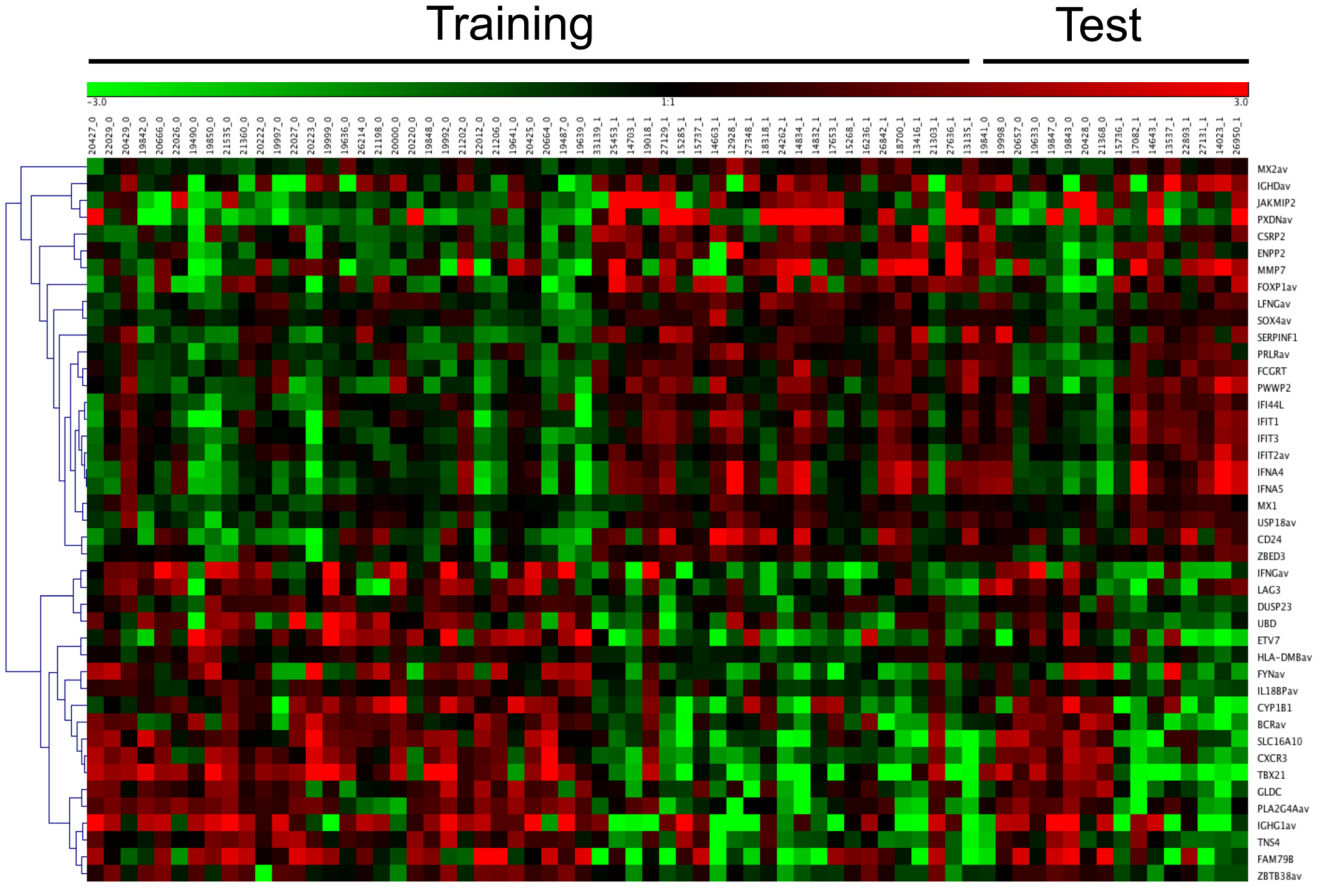
Heat map showing classification in training and test sets using the SC approach. Samples from the training set presented on the left (53) and those from the test set are presented to the right (16). *BRCA1* status is indicated at the top of the heat map; *BRCA1^+/−^* (0), *BRCA^−/−^* (1). Genes used in the predictor are listed at the right. These genes were sorted according to relatedness using the GENESIS program, and a dendrogram of relatedness is presented at the left of the figure. Samples that were mis-classified are indicated by arrows at the top of the figure. Misclassification arrows missing (as noted), remove red to green bar at top, colour code the samples to identify carriers and controls.

To graphically examine the contribution of these candidates to the classification scheme, we used the GENESIS software package to generate a heat map of expression level of these 43 genes across the cell lines tested (Fig. 1). Samples are arranged by *BRCA* status at the top, with relative expression values at the right. As expected, clustering revealed distinct expression patterns in the training set. More importantly, this clustering was clearly recapitulated in the independent test set, indicating the classification results are consistent across the 43 gene profile, rather than being driven by a small number of genes within this set.

### Genes predicting *BRCA1* status indicate defects in interferon-regulated transcriptional pathways in *BRCA1* haploinsufficient cells

To further understand the underlying biology leading to the classification scheme, we used the Ingenuity Pathway Analysis (IPA) application to explore the networks linking the genes in our predictor. IPA constructs optimal interaction networks that contain a maximum of 35 genes/proteins, and returns a graphical interaction network, as well as a calculated probability score. The probability score for any given network takes into account a number of factors, including the number of molecules on the input list that appear in the final pathway relative to random molecules in the database. The probability score for a network is calculated using a right-tailed Fisher's Exact Test [46]; and see www.ingenuity.com.

**Figure 2:**
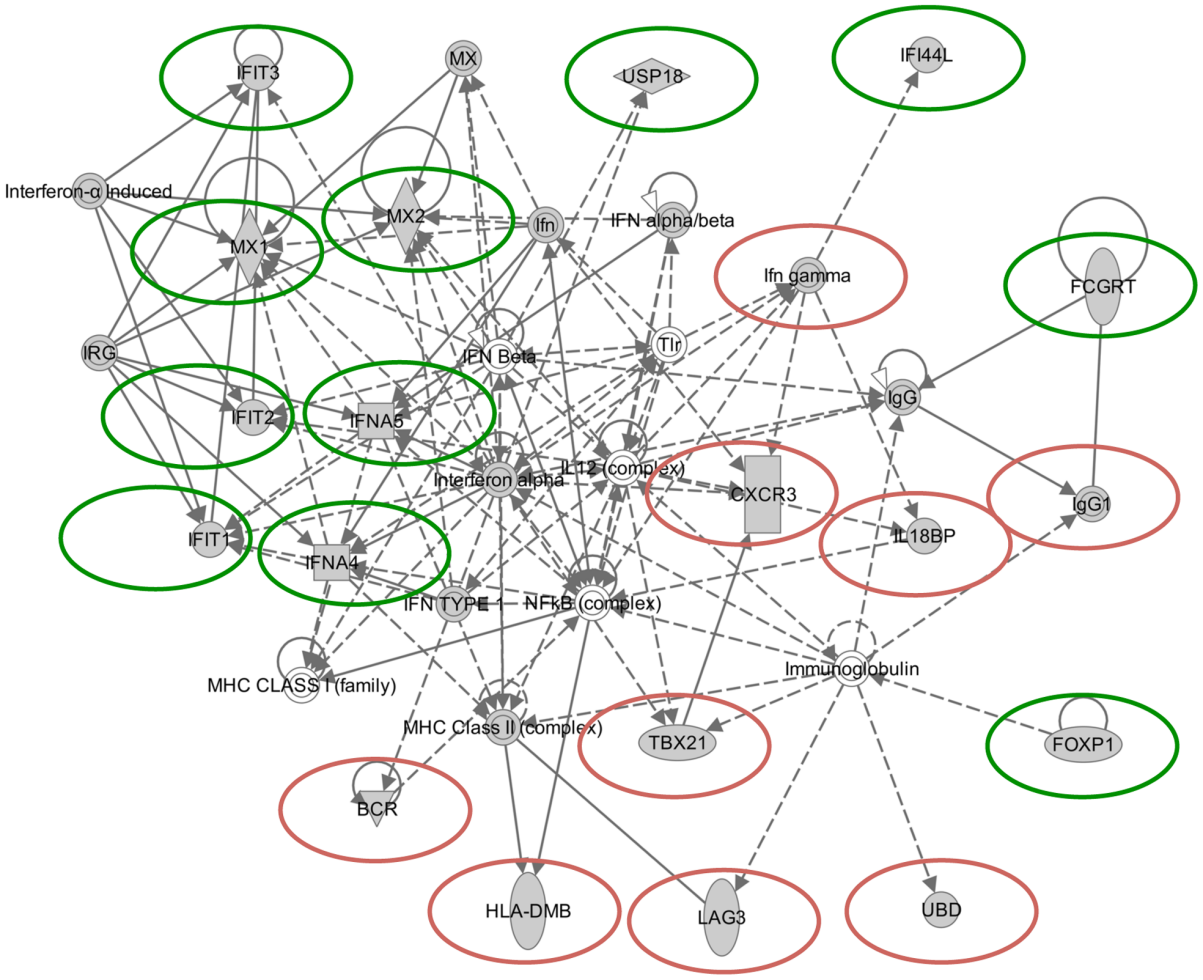
Graphical representation of the interactions of a subset of the genes identified by both the NSC and SVM approaches. A total of 22 genes were input into the Ingenuity pathway analysis program, and 11 are represented in this 35 gene output pathway. A detailed key to the analysis output can be found at https://analysis.ingenuity.com/pa/info/help/help.htm; which includes the following: Direct (solid lines) and indirect (dashed lines) Interaction; Inhibitory (bar at line end), Activating (arrow at line end) or Undefined (no line end) Binding; Regulation via Expression (E), Transcription (T) and Protein-protein interaction (P-P); Gene functions including Transcription regulators (wide ovals), cytokines (squares), complexes (double circles), enzymes (tall diamonds), and non-classified (circles).

We developed a network based on the 43 gene consensus list (Fig. 2). The network is designated as relating to “Hematological System Development and Function,” with a calculated *p*-value of 10^−26^. Of the 35 genes/complexes reported in this pathway by IPA, 20 are in our input list of 43, and an additional 9 components represent complexes containing these genes. The disease/disorder states identified as associated with the most genes in the 43 gene consensus list were cancer (26 genes, p = 5×10^−3^−7×10^−6^) and inflammatory response (22 genes, p = 5×10^−3^−7×10^−6^). The molecular and cellular functions associated with the most input genes were cellular growth and proliferation (26 genes, p = 5×10-3–2×10-6) and cellular development (23 genes, p = 5×10-3–2× 10-7). The physiological system development and functions associated with the largest numbers of input genes were hematological system development and function (21 genes, p = 5×10-3–1×10-7), and immune cell trafficking (15 genes, p = 5×10-3–1×10-7).

An alternative approach to determining the function of genes that are differentially regulated in cells derived from BRCA1 carriers is though Gene Ontology analysis. Outputs from those analyses were in general agreement with the Ingenuity-based analysis, with the top GO Process terms identified including regulation of proliferation (12 genes: CD24, CXCR3, FOXP1, FYN, IFIT3, IFNA4, IFNA5, IFNG, MMP7, PLA2G4A, SERPINF1, SOX4,) and differentiation (10 genes: CD24, CSRP2, ETV7, FOXP1, IFNA4, IFNA5, IFNG, SOX4, TBX21, UBD.) In addition, other common GO process terms found among this list were the regulation of apoptosis (11 genes, CD24, CXCR3, FYN, IFIT2, IFIT3, IFNG, MX1, PRLR, SOX4, TNS4, UBD), cytokine-mediated signaling (10 genes: IFNA5, IFNA4, IFIT1, IFIT2, IFIT3, USP18, MX1, MX2, HLA-DMB, IFNG), type 1 interferon-mediated signaling (8 genes: IFIT1, IFIT2, IFIT3, IFNA4, IFNA5, MX1, MX2, USP18), response to virus (8 genes: TBX21, MX1, MX2, IFIT1, IFIT2, IFNA4, IFNA5, IFI44L), and immune response (6 genes: PXDN, ENPP2, FCGRT, IGHD, IFI44L, IGHG1).

### Validation by qPCR

**Figure 3:**
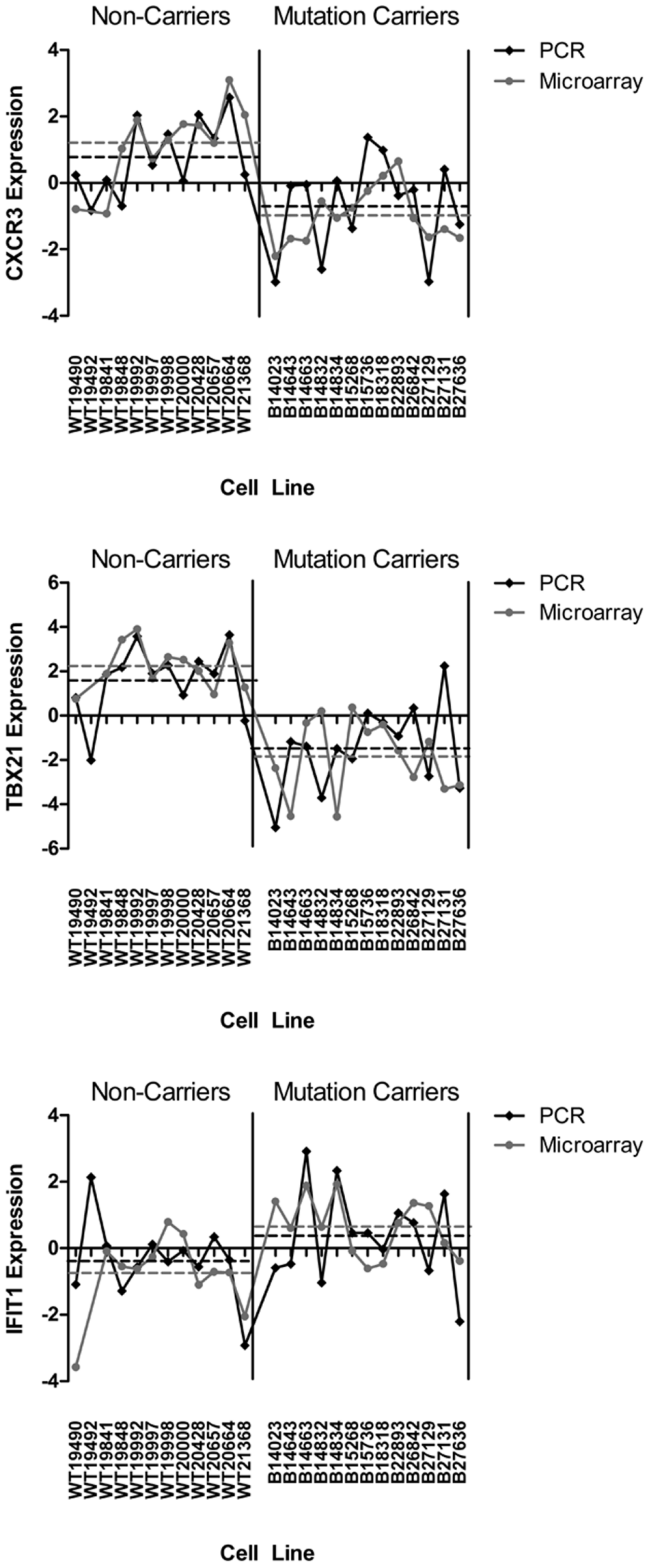
Comparison of qPCR and gene expression microarray results. Distribution of relative target expression levels between BRCA1 mutation carrier and non-carrier cell lines. Dashed lines represent mean relative expression values of each group (BRCA1 mutation carriers and non-carriers). Relative expression levels determined by microarray (black lines) and qRT-PCR (grey lines), for CXCR3, TBX21 and IFIT1.

To ensure technical reproducibility, we validated a subset of the most highly predictive genes using qPCR. RNA was prepared from the indicated control and carrier cell lines, and subjected to TaqMan assay for the indicated target genes. Data were graphed along with the gene expression microarray data obtained in the original experiment. As presented in Fig. 3, CSCR3, TBX21 and IFIT1 all reproducibly show consistency between microarray and qPCR based approaches in terms of overall average values across multiple samples, as well as a generally consistent trend between individual samples. Of the 5 genes tested in this way, 4 were shown to recapitulate the microarray data in the followup qPCR assays (Table 2).

### Differential response to radiation does not distinguish *BRCA1^+/+^* from *BRCA1^+/−^* cells

Given the association of BRCA1 with the DNA damage response, we also attempted to determine whether we could generate a molecular classifier to distinguish *BRCA1* mutant heterozygotes from wild type cells following exposure of cells to IR. Analysis was performed using nearest shrunken centroids, essentially as above, except that expression for each gene was calculated relative to its own expression level in a parallel unirradiated sample. Training set error was minimized (9%) at a single shrinkage coefficient, 1.44, representing 144 genes. However, in this case, the predictive value of the model on the independent test set was poor. Only 5 of 16 samples (31%) were correctly predicted with multiple errors in both control and carrier prediction.

To optimize the modeling, the data were filtered to remove low expressing genes, which can cause difficulty in analysis. Using the 19850 genes with highest average expression level in control samples, analysis was repeated. Again, a single shrinkage coefficient, 1.57, representing 76 genes, was found, which minimized the training set error (15%). Analysis of the independent test set yielded an accuracy of only 5 of 16 samples.

It was unclear whether the failure to generate a predictor with this dataset was because the IR treatment had not had the desired effect, or whether activation of the DNA damage response did not differentiate between BRCA1 wildtype and haploinsufficient cells. To determine whether the IR treatment had actually had the desired effect on DNA repair and checkpoint pathways, we identified the radiation-responsive genes in wild type cells, and compared these to other published datasets [45], [47], [48]. Using SAM [45], with a defined false-discovery rate of 1.5%, we identified 2643 up-regulated genes (SAM score >1.26), and 3631 down-regulated genes (SAM score <−1.16), under the conditions described above. While a complete description of these results is beyond the scope of this manuscript, about half of previously reported radiation-responsive genes were recapitulated in our data set, suggesting appropriate activation of radiation response pathways following our treatment [Table 3].

**Table 3:** Comparison of overlap between this study, and other published gene expression microarray radiation response papers.

*Study*	*#ID* '*d*	*Overlap*	*Conditions*	*Array*
Tusher (2001)	36	20	5 Gy, 4 h	6.8k features
Jen (2003)	126	50	3/10Gy, 6–24 h	12.6k features
Rieger (2004)	200	106	5 Gy, 24 h	12.6k features

Given this, we conclude that response to IR is unlikely to yield a functional discriminator for *BRCA1* haploinsufficiency, and hypothesize that manifestation of the defect in radiation response may require loss of the second BRCA1 allele.

## Discussion

Our results identify a set of dysregulated genes in unperturbed EBV-transformed lymphocytes carrying heterozygous *BRCA1* mutations. The identity of the genes in our model is consistent with the hypothesis that cells with a reduced amount of functional BRCA1 are less differentiated than non-mutant control cells. In addition, we have shown that subjecting the same cell lines to 2 Gy IR results minimal ability to discriminate cells carrying a *BRCA1* mutation from those that do not. This suggests that *BRCA1* haploinsufficiency does not cause global changes in the DNA damage response at low doses, but does not preclude such changes at higher doses. Alternatively, the DNA damage defects in *BRCA1* mutant cell lines may only be seen after loss of the wild type *BRCA1* allele. These findings together have significance for development of a functional assay for *BRCA1* carriers, as well as for understanding the biology behind *BRCA1*-dependent breast cancers.

Apart from the potential clinical significance of a rapid functional tool to identify BRCA1 heterozygous mutation carriers, our model provides intriguing data about the biological effect of haploinsufficiency of this protein. *TBX21* is one of the key genes we identified as down-regulated in *BRCA1^+/−^* LCLs compared to wildtype cells. It has been shown to act as a master regulator for T cell development, particularly via a mechanism involving interferon γ [49]. This finding is consistent with published work showing defective T cell lineage in *BRCA1* null mice [50], and that BRCA1 is involved in the development of the breast in mouse and human systems [28], [51]. In addition, breast tumors arising in *BRCA1* carriers have been shown to have increased expression of several stem cell markers [52]. Consistent with this, we found that *BRCA1^+/−^* cells exhibited down-regulation of numerous interferon-regulated genes, including *IFIT1*, *IFIT2*, *IFIT3*, *HERC5*, and *USP18*. While neither IFNβ or IFNγ were identified in our predictive models for *BRCA1* status, IFNα is a component of the optimal SVM-based predictor, further supporting the idea that interferon signaling in general may be deregulated under conditions of *BRCA1* haploinsufficiency.

Initial studies of LOH in the breast tumours of *BRCA1* carriers reported a rate of 75% (75/101) of LOH of the wild type allele [53]-[56]. More recently, quantitative allelotyping has demonstrated a significant degree of variability in the extent and direction of LOH in breast tumours [57]. In some cases, normal tissue shows LOH of the wild type *BRCA1* allele, and tumor tissue shows loss of the mutant allele.

In addition to the LOH studies, it is well established that complete loss of *BRCA1* in sporadic cancer is a very rare event [58], [59]. While this may reflect a different mechanism for *BRCA1* inactivation in sporadic cancers, such as gene silencing, it may also be that *BRCA1*-dependent oncogenesis is initially associated with haploinsufficiency during development. This may be exacerbated by the additional cancer-driving effects of homozygous *BRCA1* loss in the late stages of carcinogesis contributing to the overall aggressiveness of *BRCA1*-dependent tumours. In such a model, BRCA1 would function as both a gatekeeper (early event due to haploinsufficiency leading to reduced differentiation) and a caretaker (later effect which requires complete loss of function, contributing to aggressiveness through effects on maintenance of genomic stability). Such a dualistic role might explain the bewildering lack of consistency of the LOH studies in BRCA1-related tumours to date.

This model of BRCA1 function may also explain our results that showed a relative lack of predictive power for discriminating *BRCA1* carriers from wild type cells following irradiation. In contrast to previous finding using irradiated fibroblasts [38], [39], our results from LCLs demonstrated that IR did not accentuate differences between wild type and *BRCA1* mutation carrying cells. It is possible that these different results reflect fundamental differences in the radiation response between lymphocytes and fibroblasts.

The best model we generated was made using unirradiated cells. This is significant because it suggests that the best predictor may be achievable without the use of complicated irradiation protocols. Our model was 100% accurate in predicting the test set. There were a total of 8 samples in the training set that were not properly classified. Pooling of the test and training sets gave an overall sensitivity of 84% and a specificity of 92%. The ability to predict in an independent test set suggests that the model is robust and will likely be reproducible in further validation studies.

The accurate identification of *BRCA1* mutation carriers is an important challenge in disease management. Given the size of the gene, the heterozygous state of individuals at risk, and multiplicity of functions ascribed to the protein, it is also a significant challenge to develop a comprehensive functional assay for *BRCA1*. The results of this study indicate that *BRCA1* mutation carriers may be identified using a functional assay based on altered gene-expression profiles in non-cancerous cells. Furthermore, the ability of this assay to predict *BRCA1* status in unperturbed cells suggests that it can potentially be adapted to a simple peripheral blood-based assay. Future work will focus on determining whether the gene expression patterns seen here can be observed in fresh blood samples as well, as this would be a condition of using a variant of this assay in a clinical screening setting. In addition, future work will also focus on whether the functional data from this assay can be applied to classifying VUS alterations in BRCA1; regardless of its utility as a screening tool, such a finding would add significant clinical value by appraising the likelihood of any VUS contributing to disease risk.
